# Identification of Key Candidate Genes in Runs of Homozygosity of the Genome of Two Chicken Breeds, Associated with Cold Adaptation

**DOI:** 10.3390/biology11040547

**Published:** 2022-04-01

**Authors:** Elena S. Fedorova, Natalia V. Dementieva, Yuri S. Shcherbakov, Olga I. Stanishevskaya

**Affiliations:** Russian Research Institute of Farm Animal Genetics and Breeding—Branch of the L.K. Ernst Federal Research Center for Animal Husbandry, Pushkin, 196601 St. Petersburg, Russia; dementevan@mail.ru (N.V.D.); yura.10.08.94.94@mail.ru (Y.S.S.); olgastan@list.ru (O.I.S.)

**Keywords:** candidate genes, runs of homozygosity, SNP, cold adaptation, respiratory quotient, selection, chicken gene pool

## Abstract

**Simple Summary:**

The search for genomic regions related to adaptive abilities preserved in the chicken gene pool of two breeds, which have not been under intensive selection pressure, is of great importance for breeding in the future. This study aimed to identify key candidate genes associated with the adaptation of chickens to cold environments (using the example of the Russian White breed) by using molecular genetic methods. A total of 12 key genes on breed-specific ROH (runs of homozygosity) islands were identified, which may be potential candidate genes associated with the high level of adaptability of chickens to cold environments in the early postnatal period. These genes were associated with lipid metabolism, maintaining body temperature in cold environments, non-shivering thermogenesis and muscle development and are perspectives for further research.

**Abstract:**

It is well known that the chicken gene pools have high adaptive abilities, including adaptation to cold environments. This research aimed to study the genomic distribution of runs of homozygosity (ROH) in a population of Russian White (RW) chickens as a result of selection for adaptation to cold environments in the early postnatal period, to perform a structural annotation of the discovered breed-specific regions of the genome (compared to chickens of the Amroks breed) and to suggest key candidate genes associated with the adaptation of RW chickens to cold environments. Genotyping of individual samples was performed using Illumina Chicken 60K SNP BeadChip^®^ chips. The search for homozygous regions by individual chromosomes was carried out using the PLINK 1.9 program and the detectRuns R package. Twelve key genes on breed-specific ROH islands were identified. They may be considered as potential candidate genes associated with the high adaptive ability of chickens in cold environments in the early postnatal period. Genes associated with lipid metabolism (*SOCS3*, *NDUFA4*, *TXNRD2*, *IGFBP 1*, *IGFBP 3*), maintaining body temperature in cold environments (*ADIPOQ*, *GCGR*, *TRPM2*), non-shivering thermogenesis (*RYR2*, *CAMK2G*, *STK25*) and muscle development (*METTL21C*) are perspectives for further research. This study contributes to our understanding of the mechanisms of adaptation to cold environments in chickens and provides a molecular basis for selection work.

## 1. Introduction

The future of chicken breeding is aimed at improving the welfare of chickens, especially when the conditions for rearing chickens are as natural as possible. With such a rearing system, chickens will be exposed to various physical and climatic stresses (cold, heat and wind), infectious diseases and social stress. Additionally, birds kept in such conditions should have high adaptive abilities and stress resistance, which should not be accompanied by an increase in production costs and the risk of diseases [1].

Local breeds of chickens may have valuable genetic variants for breeding and selection of new breeds and commercial crosses in conditions of changing market demands. Local breeds of chickens are carriers of specific sets of genes responsible for adaptation to harsh environmental conditions, various diseases, etc. Advances in genetics and biotechnology make it possible to quickly transfer the unique genetic variants that have arisen in local breeds of chickens during the adaptation process to the local habitat into the genomes of commercial international high-performance breeds to preserve their outstanding properties in new habitat conditions. To increase the frequencies of adaptive alleles in commercial strains, it is also possible to use genomic selection methods [1].

Most of the research works in the field of thermoregulation genetics are devoted to adaptation to hot climate conditions, since the main areas of developed poultry farming (due to the availability of feed resources and markets—Latin America, China, India, the Middle East) are concentrated in regions with high average annual temperatures [2,3,4,5,6,7,8]. However, a significant number of chickens are housed in cold climates (Russia, Canada, Northern Europe), and significant economic costs for energy resources are necessary to maintain optimal conditions for keeping chickens. In this regard, studies aimed at identifying candidate genes involved in thermoregulation mechanisms at low temperatures are relevant.

Low temperature is the main environmental factor that limits the growth of animals and can threaten their survival [9]. Nevertheless, there are some chicken gene pools that have adapted to such harsh conditions during selection and have developed various physiological and biochemical mechanisms of adaptation to cold environments [9,10,11]. Although chickens are widespread and adapted to harsh environmental stresses prevailing in many rural areas, there are very few published studies on their adaptation to cold climates [9]. Such local breeds can be used as model populations to study the molecular genetic mechanisms of adaptation to cold environments.

One of such breeds with high adaptive abilities in low temperatures, starting from the age of one day, is the Russian White breed of chickens contained in the bioresource collection “Genetic Collection of Rare and Endangered breeds of chickens” of the Russian Research Institute of Farm Animal Genetics and Breeding-Branch of the L.K. Ernst Federal Research Center for Animal Husbandry (Genetic collection of rare and endangered breeds of chickens; available online: http://vniigen.ru/ckp-geneticheskaya-kollekciya-redkix-i-ischezayushhix-porod-kur/ (accessed on 1 February 2022)). Simultaneously, the excellent adaptability of RW chickens to cold environments in the early postnatal period has not been sufficiently studied from the viewpoint of molecular genetic mechanisms.

One of widely used areas of chip technology is genome analysis for runs of homozygosity (ROH) [12]. ROH islands are regions of chromosomes that appear in a homozygous state in the diploid genome and display identical alleles at several adjacent loci. The identification of ROH islands and potential candidate genes located on them as another useful indicator of selection signals for adaptation to cold environments was used. This study is intended to contribute to a deeper understanding of the mechanisms underlying chickens’ adaptation to such environments, which will allow a new look at adaptation to cold environments in chickens in the early postnatal period.

The main purpose of this study was to search for candidate genes associated with high adaptive abilities of chickens (*Gallus gallus domesticus*) in the early postnatal period to cold environments, using the example of a population of Russian White chickens.

## 2. Materials and Methods

### 2.1. Experimental Chicken Breeds

The object of research was chickens of the Amroks and Russian White breeds (Genetic collection of rare and endangered breeds of chickens; available online: http://vniigen.ru/ckp-geneticheskaya-kollekciya-redkix-i-ischezayushhix-porod-kur/ (accessed on 1 February 2022)) at the age of 43 weeks.

The Russian White breed was created on the basis of the White Leghorn and local breeds of chickens in the late 20th century. For neonatal chicks of this population, the conditions for normal growth and development were temperatures, on average, of 20 °C (18–23 °C) versus the generally accepted 30–33 °C [13,14].

At the initial stage of the selection work, the heterogeneity of chickens was established on the basis of their adaptive ability at low temperatures and their divergence into two alternative forms: thermoresistant and thermosensitive. The increased thermoregulatory ability, providing thermoresistance, was inherited by the dominant type. Chicks that had hypothermia in conditions of low temperatures were recessive homozygotes. Because of the individual selection of parental pairs, the thermoresistance property in the population was stabilized in subsequent generations. The dominant type of inheritance of adaptive ability at low temperatures has been confirmed by crossing RW chickens with other breeds. It was found that the property of resistance to low temperatures of day-old chicks of the new population of the RW breed is transmitted to the hybrid [13].

Physiological studies have shown that selection by the function of thermoregulation at low temperatures led to the development of biologically appropriate adaptations to these temperatures in the body, providing an energy-saving effect. RW chicks showed a decrease in the relative weight of the lungs as a mechanism for reducing heat transfer. On the first days after hatching, thermoresistant chicks in conditions of low temperatures showed a slight decrease in body temperature, which caused a slowdown in physiological processes, including respiratory function. Reducing the intensity of breathing slowed down the development of the lungs and reduced heat transfer [14].

**Figure 1:**
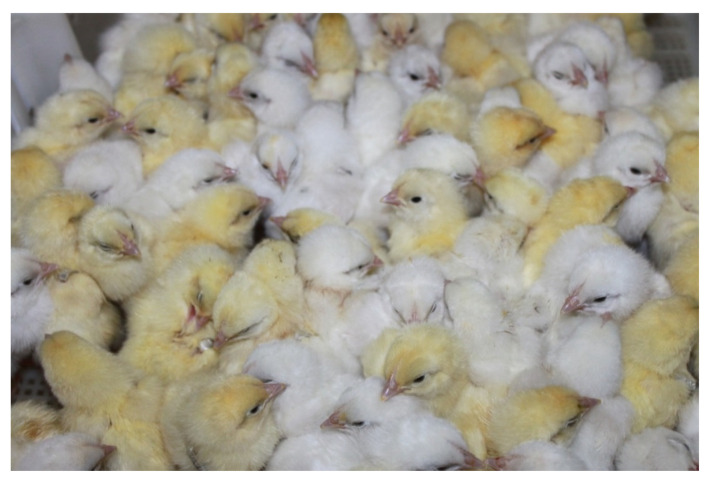
Variability of down color in day-old Russian White chicks.

Additionally, because of the selection under hypothermic stress conditions, genotypes of day-old chicks with snow-white down appeared (Figure 1).

Obviously, the selection of neonatal RW chicks by the stress response to sublethal low temperature affected the mechanism of neuroendocrine regulation of the melanogenesis process, which is controlled by pituitary, thyroid, steroid and sex hormones, resulting in a mutation in the down color of the day-old chicks. Our assumption about the influence of neuroendocrine status on the down color of neonatal chicks is confirmed by studies evaluating 10-week-old RW chicks with a snow-white down color at the day-old age, according to the degree of functional reserves of the adrenal glands detected by administrating adrenocorticotropic hormone (ACTH). It was found that the average concentration of corticosterone in the blood of chicks with a snow-white down color in response to ACTH injection was 82.6 ng/mL, while in other chicken gene pools, it was significantly lower (24.0–52.3 ng/mL) [15]. Additionally, an increase in oxidative processes associated with increased activity of the thyroid gland causes the decomposition of melanin, resulting in, for example, the white coloration of Arctic animals [16].

It is also known that one of the protective mechanisms in chicken embryos under conditions of lowering the incubation temperature is an increase in the volume of their allantois-amniotic fluid [17]. It is this mechanism that distinguishes RW chickens from chickens of other non-commercial breeds. Even with conventional incubation regimes, RW chicken embryos are characterized by a higher yield of extraembryonic fluid (mL and mL per egg weight). Although there was no selection work for the thermoresistance of RW chickens at low temperature in the period from 2000 to 2013, and the chickens were on free mating, they still retained their high adaptive ability in cold environments in the early postnatal period [18,19].

Recent studies have been devoted to the conduction of genome-wide association studies (GWAS) using single-nucleotide polymorphism (SNP) genotyping and the Illumina 60K BeadChip chip [20,21]. Several estimated associations have been related to live weight, egg weight and the yield of allantois-amniotic fluid of embryos. Additionally, an association with the snow-white down of day-old chicks was found on GGA2, where there is a non-equilibrium block for SNP coupling, including genes responsible for immune resistance [22].

**Figure 2:**
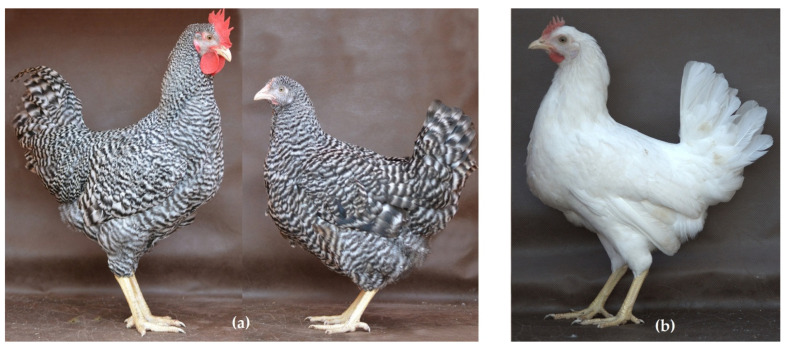
Two studied chicken breeds: Amroks (**a**) and Russian White (**b**).

The evaluation of the thermoresistance of RW chickens at low temperatures was carried out compared with chickens of the Amroks breed (Figure 2).

There was no selection work for adaptation to low temperatures in this breed. Amroks is a dual-purpose breed of chicken, and its egg production is about 200–220 eggs per year. Amroks chickens originate from the USA, specifically in Plymouth, Massachusetts. They are a crossbred of the Black Java, Black Cochin and Dominique chickens. Amroks chickens are known from 1848 as “Barred Rock Chickens”. They have the same origin as the Barred Plymouth Rock. After the Second World War in 1948, these chickens were imported to Germany, where their egg qualities and adaptability to environmental conditions were improved. The breed standard was approved in the late 1950s. The breed was named Amroks [23].

### 2.2. Experimental Design of Thermoregulation Capabilities

**Figure 3:**
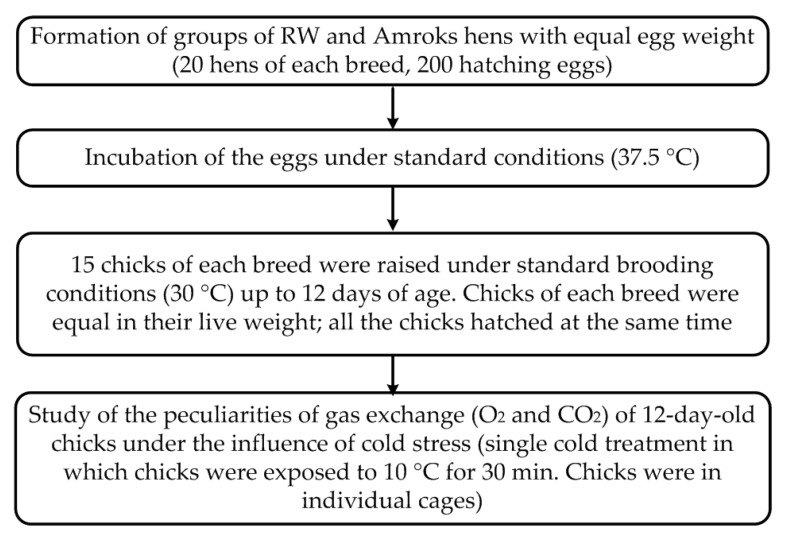
Design of the study of thermoregulation capability evaluation of RW and Amroks chicks under the influence of sublethal low temperature.

Design of the study is schematically presented in the Figure 3. Eggs were weighed on “HL-400EX” electronic scales (A&D Company Ltd., Tokyo, Japan). Hatching eggs were incubated in laboratory conditions in a “REMIL-C” incubator (REMIL, Ryazan, Russia). The gas exchange rate (oxygen consumption and carbon dioxide production) was measured using a gas analyzer, MAG-6P-K (NOVAPRIBOR, Saint-Petersburg, Russia). To measure the gas exchange, 12-day-old chicks were placed in a sealed chamber. The volume of the chamber and the exposure time of the chicks were selected to prevent hypoxia. The respiratory quotient (RQ) is defined as the volume of carbon dioxide released over the volume of oxygen absorbed during respiration. This ratio is used to indicate the mixture of lipids, carbohydrates and proteins in the metabolic substrate [24] (Figure 3).

### 2.3. Molecular Genetic Research

The material for genomic DNA extraction was blood samples taken from the vena axillaris of chickens (20 chickens of Amroks breed; 177 chickens of RW breed). Blood samples were collected by trained lab personnel following the RRIFAGB ethical guidelines for minimizing any possible bird discomfort or distress. DNA was isolated by conventional methods using phenol–chloroform extraction. The quantity and quality of DNA samples were tested on a NanoDrop 2000 spectrophotometer. Genotyping of individual samples was performed using Illumina Chicken 60K SNP BeadChip^®^ chips. All genotypes of chickens were filtered according to the effectiveness of genotyping. Only SNPs located on autosomes from GGA1 to GGA28 were used for analysis, and SNP genotypes in less than 95% of samples were excluded from the analysis. The analysis used the following parameters for filtering SNPs: --maf 0.05, --geno 0.02, --hwa 0.0001. To eliminate the effect of sex chromosomes on the subsequent estimation, SNP markers located on them were excluded.

To assess intrapopulation genetic diversity and ROH analysis, PLINK 1.9 software was used [25,26]. Additionally, DNA samples with a quality of genotyping at SNP loci of more than 95% were taken for analysis, which was performed using the Genome Studio program (“Illumina”, San-Diego, CA, USA). To eliminate the influence of gender on the assessment, SNP markers located on sex chromosomes were excluded.

The analysis of multidimensional scaling was carried out in PLINK 1.9. Visualization was conducted in R using the standard “plot” function. The Admixture v1.3 program was used to establish the population structure of the chicken groups under study, and visualization was conducted in R using the “pophelper” package [27]. To assess between-population diversity, Wright’s [28] fixation index (F_ST_) values were computed using eigensoft 6.1.4 software [29].

The search for homozygous regions by individual chromosomes was carried out using the PLINK 1.9 program and the detectRuns package in R according to the following parameters: window size, 15 SNPs; window overlap threshold, 0.1; minimum number of SNPs in the region, 15; maximum number of heterozygous SNPs in the window, −1 [30].

PLINK 1.9 was used to estimate F_ROH_ (ROH-based inbreeding coefficient with SD). The number and length of ROH for each individual were estimated and then averaged for each individual within each breed. Additionally, we calculated the coefficient of genomic inbreeding and inbreeding based on ROH (F_ROH_). The number of ROH in the genomes of the two study breeds was studied using the following ROH length classes: 0.5–1, 1–2, 2–4 and 4–8 Mb. To determine the proportion of the genome covered by different ROH segments, we calculated the sum of ROH for the following different minimum lengths: >0,5, >1, >2, >4 Mb.

The putative ROH islands were identified as overlapping homozygous regions shared by more than 50% of the analyzed individuals within each breed. A threshold of 0.5 Mb was applied for the minimum size of the overlapping length, since shorter segments of 0.5–1.3 Mb in size predominate in the genome of Russian White chickens.

To determine the prospective candidate genes in genomic regions, genomic localization of regions defined as ROH islands was used. The boundaries of these regions, localized in the chromosomes of the reference assembly GRCg6a, were used as a list of queries for extracting chicken genes and their orthologs in humans, rats and cattle using the Ensembl Genes release 103 web database and the Ensembl BioMart data analysis tool (Ensembl; available online: http://www.ensembl.org/index.html (accessed on 1 February 2022)). The adjustment of the position before the GRCg6a assembly was carried out using a file containing information about polymorphisms included in the Cobb Vantress Chicken 50 k biochip, which was provided by Illumina. The results obtained from the Ensemble Biosmart browser for each genomic region of the breeding signatures were sifted manually and compared with the corresponding published studies to determine the main candidate and other genes of interest. Additionally, with the help of Chicken QTLdb, a search was carried out for known QTL that overlap with the ROH islands identified in our studies.

Statistical processing of the results was performed in Microsoft Excel and Statistica 10.0. The average values for the groups (M) and the standard error of the averages (±SEM) were calculated. The reliability of differences was assessed by Student’s t-criterion. The differences were considered statistically significant at *p* < 0.05.

## 3. Results and Discussion

**Figure 4:**
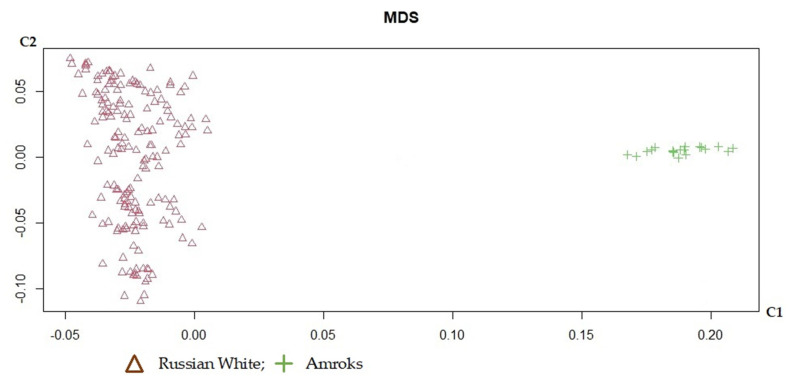
Genetic relationships among the studied chicken breeds defined through multidimensional scaling analysis.

An MDS graph to estimate the genetic relationships between the chicken breeds under study was used (Figure 4). The results of the analysis of multidimensional scaling demonstrate that both breeds (Amroks and Russian White) form non-overlapping clusters and represent clearly separate populations.

**Figure 5:**
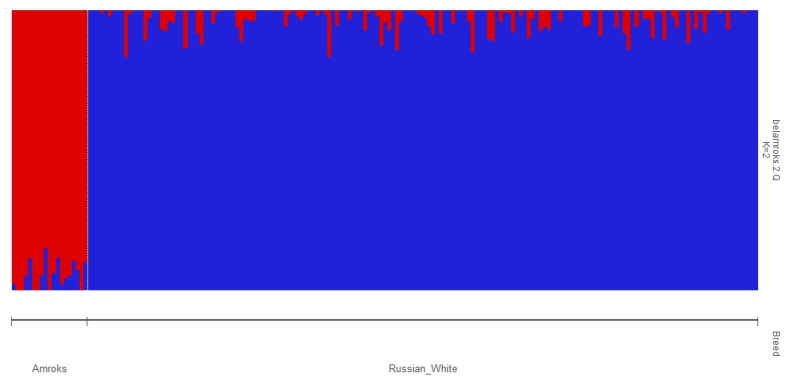
Admixture plot representing the cluster structure of the studied populations if the number of clusters K = 2.

The calculations of the CV error for the different numbers of clusters (from 1 to 5) showed that the optimal number of clusters (K) was equal to 2. The admixture clustering at K = 2 clearly distinguished the RW and Amroks populations at K = 2 (Figure 5).

The results of the genetic diversity indices are presented in Table 1. The values of expected and observed heterozygosity were higher in Amroks chickens; the distribution of MAF values was approximately uniform across the genome in both breeds; the values of the inbreeding coefficient for both breeds were almost the same.

On average, the number of ROH segments per individual was higher in the RW breed genome, which was also characterized by a lower value of the inbreeding coefficient calculated based on ROH (the difference between the breeds in terms of F_HOM_ was unreliable and not significant, so there may be inconsistent indicators with different approaches to calculations) (Table 2).

From the general database of genotypes obtained as a result of genome-wide genotyping and subsequent SNP filtering, chromosomes with the most relevant candidate genes were selected. FST (0.160 ± 0.004) was calculated for the selected chromosomes. Its value was significantly higher than in the genome-wide analysis (0.118 ± 0.0023). This is due to the peculiarities of the architecture of the genome of breeds formed under the influence of selection in the RW breed for cold tolerance in the early postnatal period.

The total length of ROH in the RW breed genome was mainly represented by a set of short segments (ROH 0.5–1.0 Mb, 61%), which indicates a low level of inbreeding in this population; the average length of ROH was 3749.6 ± 111.3 Mb per individual.

**Figure 6:**
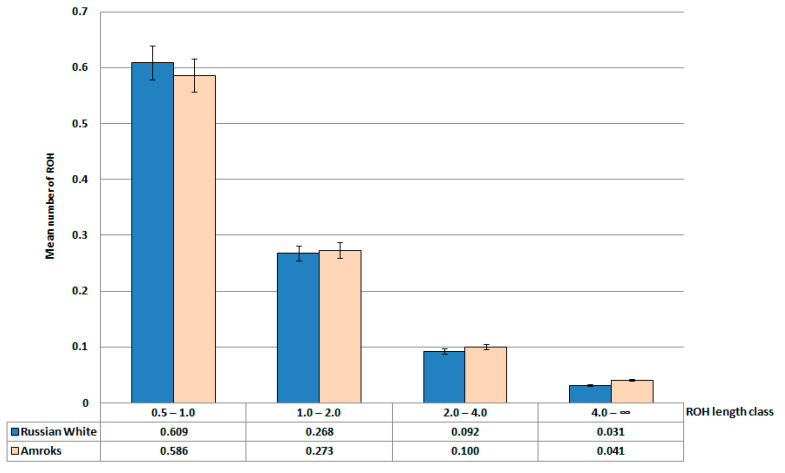
Descriptive statistics for the runs of homozygosity (ROH) by ROH length class in the breeds of Russian White and Amroks chickens: mean number of ROH (*Y*-axis) by ROH length class (*X*-axis; 0.5–1.0, 1.0–2.0, 2.0–4.0, 4.0–∞ Mb).

Descriptive statistics for the runs of homozygosity (ROH) by ROH length class in the breeds of Russian White and Amroks chickens are shown in Figure 6.

During the ROH analysis, 12 RW-specific regions on 8 chromosomes were identified, overlapping with 32 known QTL associated with plumage density, stress resistance, lung percentage, bursa, spleen and thymus gland mass, egg yolk weight, unsaturated fatty acid content in muscles, body temperature, abdominal fat mass, feed intake and blood oxygen saturation. In Amroks chickens, 5 specific regions on 4 chromosomes were found to overlap with 19 known QTL associated with egg and meat productivity, as well as reproductive qualities. Thus, the area of 50.5–54.0 Mb on GGA2 in Amroks chickens is associated with QTL for body weight at 14 and 28 days (QTL:65692 and QTL:65697, respectively) [31]; the area of 71.5–75.5 Mb on GGA4 is associated with QTL for oviduct weight (QTL:150931...150935; QTL:151098...151100) [32] and residual feed intake (QTL:195142...195144) [33]; the area of 13.8–14.1 Mb on GGA8 contains QTL for the percentage of ovaries (QTL:147743) [34].

The effect of chicken genome heterogeneity on the formation of ROH segments was also investigated by comparing ROH on macrochromosomes (GGA1 to 5), intermediate chromosomes (GGA6 to 10) and microchromosomes (GGA11 to 28). The largest number of ROH islands shared by more than 50% of the analyzed individuals was found on macrochromosomes. There were 18 regions with a length of 0.62–3.89 Mb; a total of 3 ROH islands with a length of 0.54–1.32 Mb were found on intermediate chromosomes. Six ROH were detected on microchromosomes. A total of 27 ROH islands were identified, which covered 45.71 Mb of the genome and were distributed across 12 chromosomes (GGA1, GGA2, GGA3, GGA4, GGA5, GGA6, GGA7, GGA9, GGA11, GGA13, GGA15 and GGA18). ROH islands in selected populations may indicate areas of the genome that have undergone artificial selection. In homozygous regions on 12 chromosomes of RW chickens, the presence of 404 genes associated with selection effects on the breed was revealed (225 genes were on macrochromosomes, 46 genes were on intermediate chromosomes and 133 genes were on microchromosomes).

Table 3 presents a list of 117 candidate genes located on the ROH islands, annotated in the genomes of humans, chickens and cattle, related to metabolism, immunity, development of muscle and adipose tissue and response to cold stress. Six genome regions identified on GGA2, GGA5, GGA7, GGA11 and GGA13 were common to both analyzed chicken breeds; twelve ROH were specific to RW chickens. To search for key candidate genes related to adaptation to cold environments, the sample was limited to ROH islands specific to RW chickens, since the thermal resistance selection was carried out only in this breed. It is suggested that ROH segments, which overlap in both breeds, reflect the influence of selection pressure and contain genes and their variants associated with egg production, as well as with reproductive ability.

The well-known QTL found using Chicken QTLdb in the regions common to Amroks and RW chickens confirm this assumption. A total of 6 regions on 5 chromosomes were identified, common to both study breeds, overlapping with 16 known QTL. Selective changes mainly covered QTL for growth, egg productivity, egg quality and reproductive qualities of chickens, since selection work for increasing productive rate indicators in both breeds was carried out. Thus, the regions 48.88–48.93 Mb on GGA2 and 2.14–4.51 Mb on GGA5, because of selection pressure in the RW and Amroks breeds, were associated with multiple QTL for live weight at 21 and 28 days (QTL:95404 and QTL:95415, and QTL:95416, respectively) [35]. The area of 13.18–14.51 Mb on GGA7 overlapped with QTL for eggshell strength (QTL:214446) [36], Haugh units (QTL:16765) [37] and ovarian mass (QTL:147742). On GGA11, two areas common to both breeds were identified, overlapping QTL for feed consumption (QTL:64559, 2.35–2.62 Mb, and QTL:64560, 10.20–10.82 Mb) [38], egg production (QTL:135688, 2.35–2.62 Mb) [39] and abdominal fat mass (QTL:36071, 10.20–10.82 Mb) [40]. The area on GGA13 of 4.43–5.43 Mb was associated with QTL for spleen mass (QTL:21732, QTL:21771) [41], weight and percentage of testes (QTL:147740 and QTL:147741, respectively) and oxygen partial pressure as a reaction to heat stress (QTL:71141) [42].

Twelve prioritized candidate genes on breed-specific ROH islands (Table 3) were identified, which may be potential candidate genes associated with the high adaptive ability of RW chickens in cold environments.

The study of the mechanisms of thermoregulation of RW and Amroks chicks in the early postnatal period showed that RW chicks have higher adaptive abilities in low-temperature conditions. Amroks chicks under the influence of cold stress maintain their temperature homeostasis mainly due to the consumption of lipid reserves of the body. This was evidenced by a decrease in the value of the RQ by 3.2% (Table 4 and Table 5).

RW chickens under the influence of cold stress maintain their temperature homeostasis mainly due to the consumption of carbohydrate reserves of the body. This was evidenced by an increase in the value of the RQ by 3.0% (Table 4 and Table 5). Obviously, selection for adaptation to a cold environment led to a decrease in the sensitivity of central thermoreceptive structures to low temperatures and an increase in the threshold for the occurrence of a reaction to cold. For RW chicks, cold exposure of such a level (30 min at +10 °C) is not stressful (Table 5). The differences in the starting level of the RQ between the breeds are explained by the higher relative lung mass of RW chickens (*p* < 0.05) and, accordingly, a higher level of oxygen consumption.

In this regard, the key candidate genes found on RW-specific ROH islands associated with lipid metabolism (*SOCS3*, *NDUFA4*, *TXNRD2*, *IGFBP1*, *IGFBP3*), maintaining body temperature in cold environments (*ADIPOQ*, *GCGR*, *TRPM2*), non-shivering thermogenesis (*RYR2*, *CAMK2G*, *STK25*) and muscle development (*METTL21C*) are essential (Table 6).

## 4. Conclusions

A search for genomic regions associated with the selection of chickens for adaptation to cold environments was performed. Twelve ROH islands specific to RW chickens, five ROH islands specific to Amroks chickens and six ROH islands common to both breeds were found. Several identified genomic regions overlapped with known QTL responsible for the egg production, egg quality, growth and reproductive traits of chickens. This reflects the long-term selection of chickens of both breeds to increase their productive traits.

A set of 117 candidate genes localized in selected regions of the genome was determined, as well as these genes’ functional significance. As a result, 12 of the most relevant candidate genes, associated with the cold tolerance of chickens in the early postnatal period, were identified.

Candidate genes associated with lipid metabolism (*SOCS3*, *NDUFA4*, *TXNRD2, IGFBP 1*, *IGFBP3*), maintaining body temperature in the cold (*ADIPOQ*, *GCGR*, *TRPM2*), non-contractile thermogenesis (*RYR2*, *CAMK2G*, *STK25*) and muscle development (*METTL21C*) are of the greatest interest.

This study contributes to our understanding of the mechanisms of thermal resistance in chickens in the early postnatal period and provides a molecular basis for selection work in this field.

## Figures and Tables

**Table 1:** Genetic diversity indices for the analyzed Russian White and Amroks chicken breeds.

Population	*n*	Ho ± SD	He± SD	MAF ± SD	F_HOM_ ± SD
Russian White	177	0.285 ^a^ ± 0.001	0.296 ^a^ ± 0.001	0.270 ^a^ ± 0.001	0.020 ± 0.016
Amroks	20	0.357 ^b^ ± 0.004	0.353 ^b^ ± 0.001	0.223 ^b^ ± 0.001	0.017 ± 0.005

Abbreviations: F_HOM_, inbreeding coefficient; He, expected heterozygosity; Ho, observed heterozygosity; MAF, average minor allele frequency; N, the number of individuals per breed. Values superscripted by different letters within the same column are significantly different (*p* <0.001).

**Table 2:** Descriptive statistics for ROH of Russian White and Amroks chicken breeds.

Population	*n*	F_ROH_ ± SD	MN_ROH_ ± SD
Russian White	177	0.083 ^a^ ± 0.006	25.9 ^a^ ± 1.1
Amroks	20	0.105 ^b^ ± 0.009	18.9 ^c^ ± 1.5

Abbreviations: F_ROH_, mean ROH-based inbreeding coefficient with SD; MN_ROH_, mean number of ROH per individual; N, the number of individuals per breed; ROH, runs of homozygosity. Values superscripted by different letters within the same column are significantly different (ab at *p* < 0.05; ac at *p* < 0.001).

**Table 3:** Annotated candidate genes in RW chickens, presumably associated with their adaptation to low temperatures (genes in bold type are the most relevant candidate genes).

GGA	Region (Mb)	Breed	Genes
1	18.07…20.85	RW	*CRELD2, ALG12, MLC1, IL17REL, TRABD, MAPK11, MAPK12, PLXNB2*
	143.35…145.21	RW	*SLC10A2*, ***METTL21C***, *FGF14, NALCN*
2	26.01…27.01	RW	***NDUFA4***
	47.88…48.93	RW	*FKBP9, PDE1C, PPP1R17, NEUROD6*
		Amroks	
	54.86…56.83	RW	***IGFBP1**, **IGFBP3**, NFX1, LRRC14B, RECK, RBFA, HSBP1L1, NFATC1, HNF4G, IL7*
	122.18…126.07	RW	*WWP1, ATP6V0D2, DECR1, RIPK2, OSGIN2, NECAB1, RUNX1T1*
3	36.71…39.95	RW	***RYR2**, MIR, GPR137B, ERO1B, EDARADD, LGALS8, GGPS1,COA6, DISC1*
	81.27…84.12	RW	*CD109, CGAS, MTO1, OGFRL1, B3GAT2, FAM135A*
4	17.82…19.76	RW	*RNF128, HMGB3, MTM1*
5	2.14…4.51	RW, Amroks	*PRMT3*, *NELL1, ANO5, SLC17A6, GAS2, SLC5A12, FIBIN, LGR4, BDNF*
6	16.65…17.20	RW	***CAMC2G***, *MYOZ1, SORBS1*
7	13.18…14.51	RW, Amroks	*CTLA4, ICOS, CD28, NBEAL1, IDH1, PIKFYVE, PPP1R1C*
9	5.13…5.88	RW	*HDLBP*, ***STK25**, ETV5, DGKG*, ***TRPM2***, ***ADIPOQ**, GPR55, CAB39, HES6, PIK3CB*
11	2.35…2.62 10.20…10.82	RW, Amroks	*HSF4*, *MBTPS1*, *NECAB2* *CEBPG, KCTD15*
13	4.43…5.43	RW, Amroks	*KCNIP1, KCNMB1*, *LCP2, DOCK2*
15	1.30…2.61	RW	***TXNRD2***, *COMT*, *ZDHHC8*, *DGCR8*, *PGAM5*
18	9.13…10.86	RW	*TSPAN10, SLC38A10, NPTX1, USP36, ENGASE, **SOCS3**, TK1, TMC6, AFMID, **GCGR**, SIRT7, RYCR1, SPAG9, NPB, CACNA1G, WFIKKN2, ACSF2, CD300LG, LRRC59, FADDS6, MIF4CD, KCTD2*

**Table 4:** Changes in respiratory quotient as a result of cold stress in 12-day-old chickens of the RW and Amroks breeds.

Environmental Conditions	Russian White	Amroks
Number of chicks	15	15
Standard rearing conditions (30 °C) before cold treatment	0.858 ^a^ ± 0.01	0.913 ^a^ ± 0.01
Immidiatelly after 30 min of cold treatment (at 10 °C)	0.884 ^b^ ± 0.009	0.884 ^b^ ± 0.01

Values superscripted by different letters within the same column are significantly different (*p* < 0.05).

**Table 5:** Changes in behavioral reactions and the level of gas exchange in RW and Amroks chicks under the influence of cold stress.

Parameters	Russian White	Amroks
Number of chicks	15	15
Huddling, muscle shivering	no	yes
Torpor (in individual cages)	no	yes
Changing the oxygen consumption level, %	+10.9	+15.6
Changing the carbon dioxide output level, %	+13.5	+13.5
Changing the respiratory coefficient level, %	+3.0	−3.2

**Table 6:** Characteristics of the most relevant candidate genes in RW chickens, associated with adaptation to cold environments.

Gene	GGA	Function
* SOCS3 *	18	In the adipocytokine signaling pathway, *SOCS3* affects adipogenesis by inhibiting the expression of *LERP* (lipoforin receptor) and *IRS1* (insulin receptor), participating in the adipocytokine signaling pathway. *IGF2BP3* affects the insulin signaling pathway by regulating intramuscular fat deposition [43]. In mammals, the immune system takes the most active part in the transformation of one adipose tissue into another. White adipose tissue macrophages force fat cells to turn into brown adipose tissue cells when the temperature drops. During physical exercise and when the ambient temperature decreases, meteorin-like hormone is released from the muscles, which, through the immune signaling proteins interleukins, acts on macrophages located in adipose tissue. Then, under the action of special signaling proteins, macrophages compel adipose tissue to burn its reserves. *SOCS* proteins also regulate innate immunity cells [44]. Exposure to cold causes hyperphagia to counteract fat loss associated with lipid mobilization and thermogenic activation. In experiments, chronic cold stress increased the expression of *SOCS3* gene mRNA in the hypothalamus and peripheral mononuclear blood cells in rats and ferrets [45]. The selected ROH island overlapped with QTL for metabolic traits: the level of total protein in the blood (QTL:165737) [46], as well as with QTL for immune traits: bursa mass (QTL: 101247) [47].
RYR2	3	High-conductivity Ca^2+^ release channels, known as ryanodine receptors (RyR), mediate the release of Ca^2+^ from the endo/sarcoplasmic reticulum. Prolonged exposure of birds to cold causes the development of non-shivering thermogenesis (NST) of muscular origin. NST is characterized by increased heat release, which can be achieved by increasing the ATP-dependent Ca^2+^ cycle between the sarcoplasmic reticulum (SR) and cytosolic compartments in the muscles. Studies on the effect of prolonged cold exposure on SR function in ducklings have established that the activity of SR Ca^2+^-ATPase, and the proportion of vesicles containing a Ca^2+^ release channel sensitive to ryanodine in the *muscle gastrocnemius* of ducklings, increased by 30–50% in response to prolonged exposure to cold. These results show that the content of two components directly involved in the Ca^2+^ cycle by SR increases with acclimatization to cold, and this is due to NST [48]. The region of *RYR2’s* location found as affected by selection in WC chickens overlaps with several QTL associated with the percentage of intramuscular fat (QTL:24481) [49], as well as with the pH of the pectoral muscle (QTL:157164), which reflects the glycogen content in chicken muscles and is associated with diseases of glycogen accumulation in humans [50].
* CAMK2G *	6	This gene participates in the transport of Ca^2+^ in the sarcoplasmic reticulum in skeletal muscles. In slow-twitch muscles, it participates in the regulation of Ca^2+^ transport in the sarcoplasmic reticulum (SR), and in fast-twitch muscles, it participates in the control of Ca^2+^ release from SR by phosphorylation of the ryanodine receptor. The studied region of the genome overlaps with several QTL associated with the fatty acid composition of meat: QTL for the content of stearic acid (QTL:193105), oleic acid (QTL:193106) and linoleic acid (QTL:193113) [51]. The skeletal muscles of birds have a very high ability to absorb circulating fatty acids. Endurance muscle work in birds can actually be provided with up to 95% of the energy derived from lipids. The use of this high-calorie fuel allows birds to successfully activate the mechanisms of NST under the impact of cold stress [52].
* NDUFA4 *	2	This gene is a component of cytochrome-c-oxidase, the last enzyme in the mitochondrial electron transport chain that controls oxidative phosphorylation. Cytochrome-c-oxidase is a component of the respiratory chain that catalyzes the reduction of oxygen to water [53]. According to literature data, in skeletal muscles of cold-adapted chickens, there is an increase in the content of slow-twitch muscle fibers that contain many mitochondria and use oxidative phosphorylation to produce ATP [54,55].
* METTL21C *	1	This gene is closely related to the development of chicken muscles. *METTL21C* performs regular processing of the protein structure and is also a heat shock protein that acts as an ATP-dependent molecular chaperone. *METTL21C* can regulate calcium homeostasis in bones and muscles and promote differentiation of myoblasts into muscle tubes, as well as reducing osteocyte apoptosis and regulating intracellular homeostasis [56]. The studied RW-specific ROH island with the *METTL21C* gene located is also overlapped by the well-known QTL for the pH of the pectoral muscle (QTL:157161), reflecting the glycogen content in the muscles, which, in turn, affects the processes of shivering thermogenesis.
* TXNRD2 *	15	Thioredoxins are crucial for the redox regulation of protein function and signal transmission through the redox control of thiols. Mammalian cytosolic thioredoxin performs many functions to protect against oxidative stress and controls growth and apoptosis, but it is also secreted and has co-cytokine and chemokine activities [57].
* IGFBP1,3 *	2	This gene can regulate the expression of genes related to the metabolism of fatty acids and promote the proliferation and differentiation of adipocytes [58].
* STK 25 *	9	This gene is crucial for regulating glucose and insulin homeostasis in the body and accumulation of ectopic lipids. *STK25* interacts with *GOLPH3* and mediates glycolysis through *GOLPH3*-regulated mTOR signaling [59]. Partial depletion of *STK25* increases the expression of uncoupling protein 3 (Ucp3), which is accompanied by increased lipid oxidation in myoblasts. Additionally, a reduced level of *STK25* indirectly improves insulin-stimulated glucose uptake by muscle cells [60].
*ADIPOQ*	9	This gene is involved in the differentiation of adipocytes in mammals and may play a similar role in chickens. The structures of the adiponectin protein domain among the homologs of chickens and mammals are highly conserved [61]. Adipose tissue can secrete hormones called adipokines, which play an important role in regulating metabolic and reproductive processes at both the central and peripheral levels. The metabolic system of chickens is closely related to that of mammals. Glucose is stored in tissues as glycogen and is used for energy production through glycolysis. However, chickens are constantly hyperglycemic and insulin-resistant, which mimics the condition of type 2 diabetes in mammals. Glycemic levels depend on the line, age and sex of birds, and increased obesity in chickens is associated with lower fasting plasma glucose levels, which contrasts with the situation in mammals [62]. Adiponectin is an important regulator of thermogenesis, and it is necessary to maintain body temperature when exposed to cold [63]. Adiponectin is a circulating hormone that is predominantly produced by adipose tissue. Several pharmacological studies have shown that this protein has powerful antidiabetic, antiatherogenic and anti-inflammatory properties. *ADIPOQ* plays a moderate regulatory role in mitophagy caused by oxidative stress and suppresses apoptosis. These data demonstrate the antioxidant potential of *ADIPOQ* in skeletal muscle diseases associated with oxidative stress [64].
* GCGR *	18	A glucagon-like peptide (*GCGL*) encoded by a glucagon-like gene identified in chickens and other lower vertebrates is probably a hypophysiotropic factor in non-mammalian vertebrates. Hypothalamic *GCGL* is a thyroid-stimulating hormone release factor active in chickens and participates in the control of the pituitary–thyroid axis [65].
* TRPM2 *	9	This class of ion channels, which belongs to the superfamily of transient receptor potentials (*TRP*) and is present in specialized neurons that are projected onto the skin, evolved as temperature sensors. *TRP* thermal channels are polymodal receptors. That is, they can be activated by temperature, voltage, pH and chemical agents [66]. *TRPM2* initiates a “warm” signal that controls the coolness search behavior. In this regard, it should be noted that the behavioral reactions of neonatal RW chicks under standard conditions of rearing (+30–32 °C) differ markedly from the reaction of chicks of other breeds: they tend to look for a zone with a lower temperature. In the absence of such an opportunity, they try to cool off by bathing in a drinking bowl. *TRPM2* is involved in stress-related inflammatory, vascular and neurodegenerative conditions [67]. *TRPM2* is a member of the melastatin-related transient potential ion channel receptor subfamily associated with inflammatory and neuropathic pain perception. The main endogenous activator of the *TRPM2* channel is ADP-ribose, which has been identified in *TRPM2* as a high-temperature sensor, which plays a central role in preventing overheating when the temperature rises. In addition, some reactive oxygen species are positive regulators of *TRPM2* activation; thus, this ion channel plays an important role as a sensor of the oxidative response in some cellular systems [68]. Additionally, *TRPM2* plays a key role in the development of pathological pain. Thus, in mice deprived of *TRF2* expression, the pain response decreased [69]. This gene is supposed to have an effect on reducing the sensitivity of central thermoreceptive structures to low temperature in RW chickens, which contributes to the adaptation of the body in cold environments.

## Data Availability

Chicken genotypes used during the current study are available from the corresponding author upon reasonable request. The list and location of candidate genes that were found in this study are provided in Table 3 in this manuscript.
